# Supplementary material for: A proteogenomic signature of age-related macular degeneration in blood
Source: Nat Commun. 2022 Jun 13;13:3401. doi: 10.1038/s41467-022-31085-x (PMC9192739; doi:10.1038/s41467-022-31085-x)
Supplement: Supplementary file 1 — Supplementary Information [file 41467_2022_31085_MOESM1_ESM.pdf]

Supplementary Information

A Proteogenomic Signature of Age-related Macular Degeneration in Blood

Valur Emilsson[1,2,*], Elias F. Gudmundsson[1], Thorarinn Jonmundsson[1], Brynjolfur G. Jonsson[1], Michael Twarog[3], Valborg Gudmundsdottir[1,2], Zhiguang Li[4], Nancy Finkel[3], Stephen Poor[3], Xin Liu[3], Robert Esterberg[3], Yiyun Zhang[3], Sandra Jose[3], Chia-Ling Huang[3], Sha-Mei Liao[3], Joseph Loureiro[3], Qin Zhang[3], Cynthia L Grosskreutz[3], Andrew A Nguyen[3], Qian Huang[3], Barrett Leehy[3], Rebecca Pitts[3], Thor Aspelund[1], John R. Lamb[5], Fridbert Jonasson[2,6], Lenore J. Launer[4], Mary Frances Cotch[7], Lori L. Jennings[3], Vilmundur Gudnason[1,2] and Tony E. Walshe[3,*]

[1]Icelandic Heart Association, Holtasmari 1, IS-201 Kopavogur, Iceland.
[2]Faculty of Medicine, University of Iceland, 101 Reykjavik, Iceland.
[3]Novartis Institutes for Biomedical Research, 22 Windsor Street, Cambridge, MA 02139, USA.
[4]Laboratory of Epidemiology and Population Sciences, National Institute on Aging, MD, USA.
[5]Novartis Institutes for Biomedical Research, 10675 John Jay Hopkins Drive, San Diego, CA 92121, USA.
[6]Department of Ophthalmology, University Hospital, Reykjavik, Iceland.
[7]Division of Epidemiology and Clinical Applications, National Eye Institute, National Institutes of Health, Maryland, USA.

*Correspondence: valur@hjarta.is and tony.walshe@agios.com

Supplementary Note 1

Proteins associated with AMD-linked genetic variants map to core pathways involved in AMD pathobiology.

In a recent review, a comprehensive pathway map of AMD based on 110 high-risk candidate genes, was presented[1]. Many of the proteins linked to AMD-associated variants in the present study map to these pathways, including CFH, C2, CFB, C3, CFI, PILRA and PRLR (Supplementary Data 10), all of which are involved in para-inflammation homeostatic processes. Others include MUC1, TIMP3, and TNXB, which are involved in extracellular matrix (ECM) homeostasis, VEGFA, involved in choroidal homeostasis, and ICAM3, in phagocytosis (Supplementary Data 10). VEGFA, a promoter of neo-angiogenesis, for example, is increased in the ocular tissues of nAMD patients[2]. Drusen, accumulating under the retina in AMD, are comprised of lipoprotein particles and a variety of proteins[3], including TIMP3, CLU, VTN, in addition to complement system proteins[4-6], many of which are associated with the AMD-associated risk variants studied here (Supplementary Data 7-9). For example, the *CETP* intron variant rs1864163 was associated with nine proteins, two of which were apolipoproteins (APOA5 and APOM), while rs17231506 (upstream of *CETP*, $r^2 = 0.14$ with rs1864163) associated with 10 proteins, three of which were apolipoproteins (APOA5, APOM and APOC3). Similarly, and beyond complement, variant rs62358361 (allele G), located in an *C9* intron, which is protective for AMD, was associated with a significant reduction in CREBBP levels – the master regulator of hepatocyte lipid metabolism, implicating a potential role for liver metabolism pathways in AMD. These findings are consistent with the response to retention hypothesis of AMD[7]. The AMD variant rs570618 in the *CFH* gene regulates CLU (Supplementary Data 8), which is one of the most prominent proteins in drusen, and it is substantially increased in advanced AMD[8]. When the top and bottom quintiles for CLU were compared to different AMD

outcomes, we found that serum CLU levels were highest in AMD with GA ($\beta = 0.296$, $P = 0.001$), which is consistent with increased levels associated with the rs570618 T risk allele. It should be noted that the AMD risk allele for the rs570618 variant was not linked to higher levels of complement activation in AMD patients[9], implicating non-complement pathways associated with this variant (Supplementary Data 10), or increased complement activity at ocular cell surfaces such as Bruch's membrane or choriocapillaries[9]. Inflammation and recruitment of microglia cells have been implicated in the pathophysiology of AMD, specifically retinal neovascularization[10], but the proteins involved are unknown. The microglia associated proteins TREM2 and AIF1 were found to be associated with the AMD risk variant and *trans* hotspot rs11080055 in the *VTN/TMEM97* locus (Supplementary Data 9). The serum level of the microglia and macrophage activity biomarker TREM2[11], previously linked to Alzheimer's disease[12], was positively associated (comparing the top and bottom quintiles) with nAMD but not GA (nAMD $\beta = 0.403$, $P = 1.2\times10^{-5}$; GA AMD $\beta = 0.156$, $P = 0.157$), implying increased macrophage (and/or microglia) activity associated with neovascularization in AMD patients' retinas. AIF1 serum levels, like TREM2, were positively associated (comparing the top and bottom quintiles) with an increased risk of AMD but predominantly early AMD ($\beta = 0.08$, $P = 0.007$), and through logistic regression using the entire sample (Supplementary Data 15). Notably, AIF1 was found to be causally related to AMD in the extended MR analysis, with the causal estimate being consistent with the observational estimate (Supplementary Fig. 11, Supplementary Data 15).

Proteins not previously implicated in the pathophysiology of AMD included five proteins that were associated with rs72802342 in the *CTRB2/CTRB1* locus, enriched for pancreatic secretion (KEGG database, $P = 1.4\times10^{-8}$) (Supplementary Data 10). No other proteins were linked to this

variant. These five proteins have been associated with pancreatitis and/or pancreatic cancer and breakdown of the ECM[13-16]. In passing, ECM pathway dysregulation is a known risk factor for AMD[17]. Finally, we find that the 340 proteins associated with by AMD risk loci (Supplementary Data 8), were over-represented in the serum protein network PM13 and the interconnected larger cluster of modules PM11, PM14 and PM15 (Supplementary Data 12). The inclusion of proteins regulated by the *trans* hotspot rs11080055, located at the *VTN/TMEM97* locus, in the immune and inflammation related cluster of modules has been previously reported[18]. It should be noted though, that rs11080055 only accounts for a very small portion of AMD's genetic liability (Supplementary Data 7). In summary, the findings highlighted above indicate that serum proteins link genetics to the central molecular pathology of AMD in the retina.

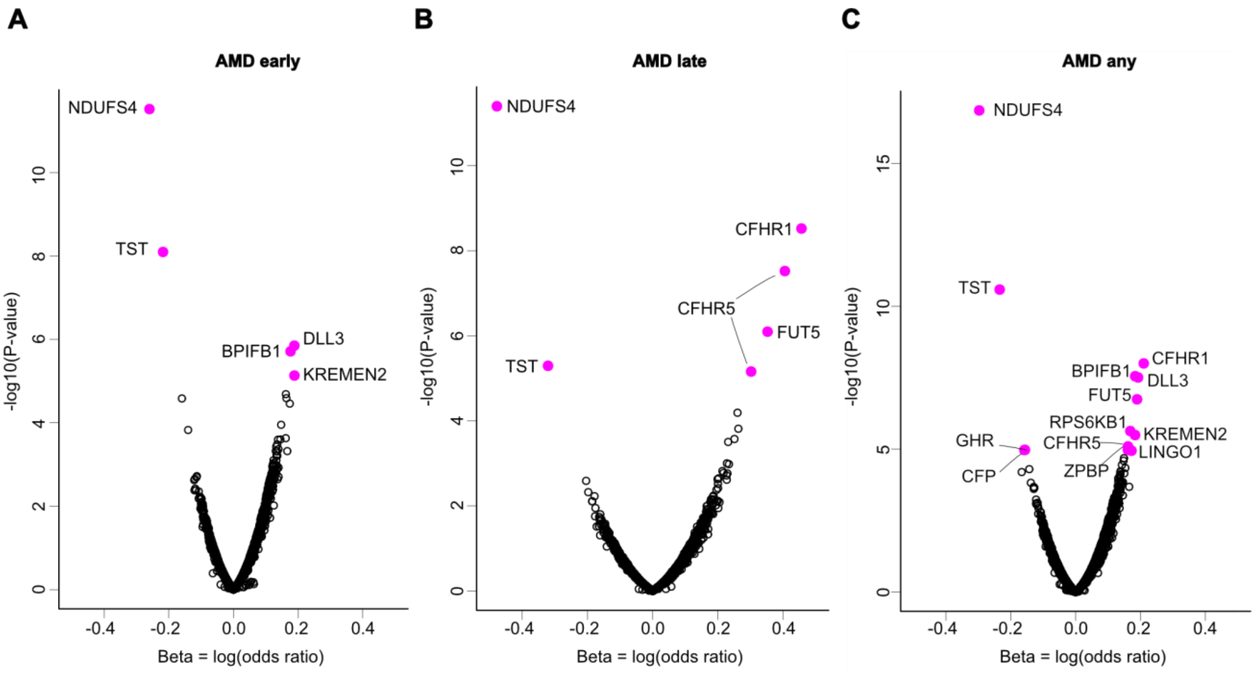

Supplementary Fig. 1. **Association of global serum proteins with different stages of AMD outcome. A**. Using logistic regression analysis and Bonferroni correction for multiple comparisons a volcano plot of all serum proteins associated with AMD early is shown. Definition of early AMD was according to Jonasson et al.[19]. **B**. A volcano plot of late AMD, and **C**. A volcano plot of AMD any, where early stage of the disease wase defined according to Jonasson et al.[19]. Colored data points highlight study-wide significant associations (P-value < 1.2×10^{-5}, two-sided). The y-axis represents the -log of P-values (two-sided) for the logistic regression analyses, while the x-axis represents the estimate as beta-coefficients equal to log(OR).

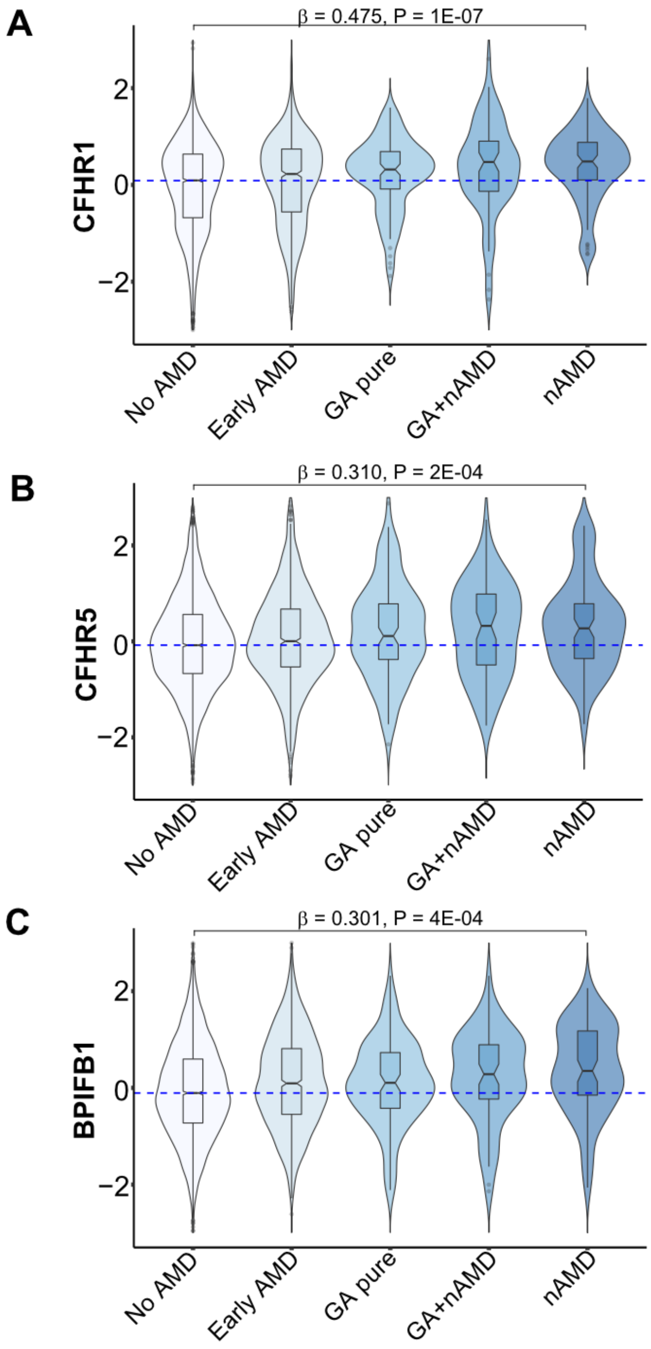

Supplementary Fig. 2. **Protein levels measured at baseline changing from no AMD to advanced AMD**. The violin plots show a near steady rise in levels of **A**. CFHR1, **B**. CFHR5 and **C**. BPIFB1 from no AMD to early AMD (definition by Jonasson et al.[19]) to advanced nAMD. The beta = log(OR) and P-values (two-sided) at the top of each plot are the results of a logistic regression analysis (adjusting for age and sex) comparing those with no AMD to those with nAMD. The boxplot indicates median value, 25th and 75th percentiles. Whiskers extend to smallest/largest value no further than 1.5 × interquartile range with outliers being shown. The number of patients in each AMD-related group in the AGES-RS cohort is shown in Supplementary Data 1 and highlighted in Methods.

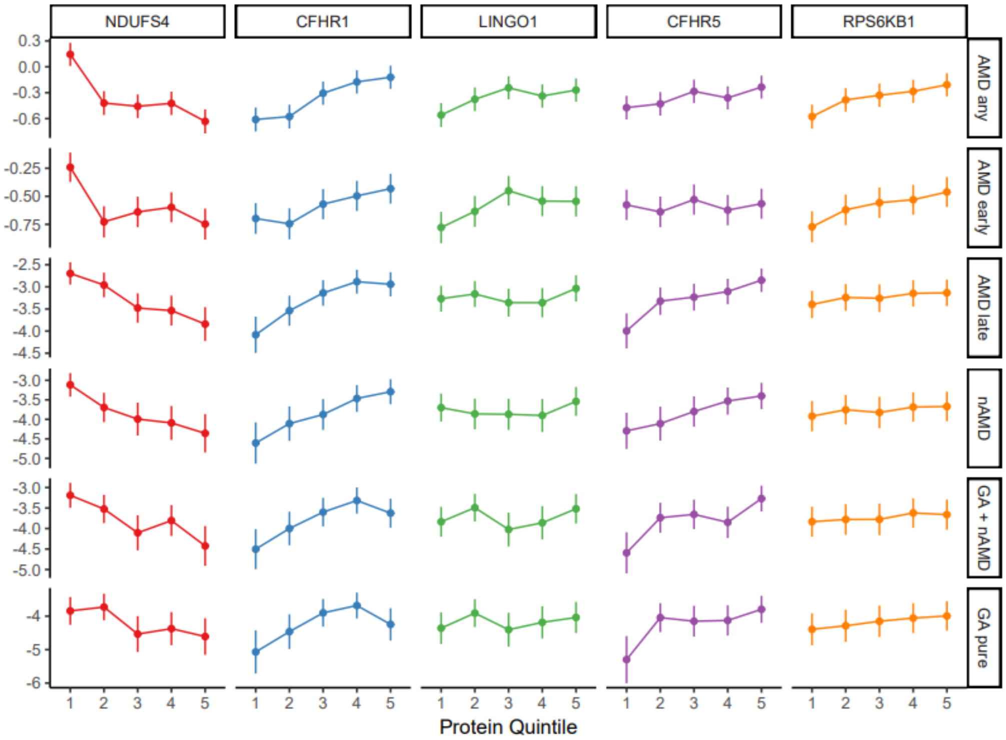
NDUFS4
CFHR1
LINGO1
CFHR5
RPS6KB1
AMD any
AMD early
AMD late
nAMD
GA + nAMD
GA pure
Protein Quintile

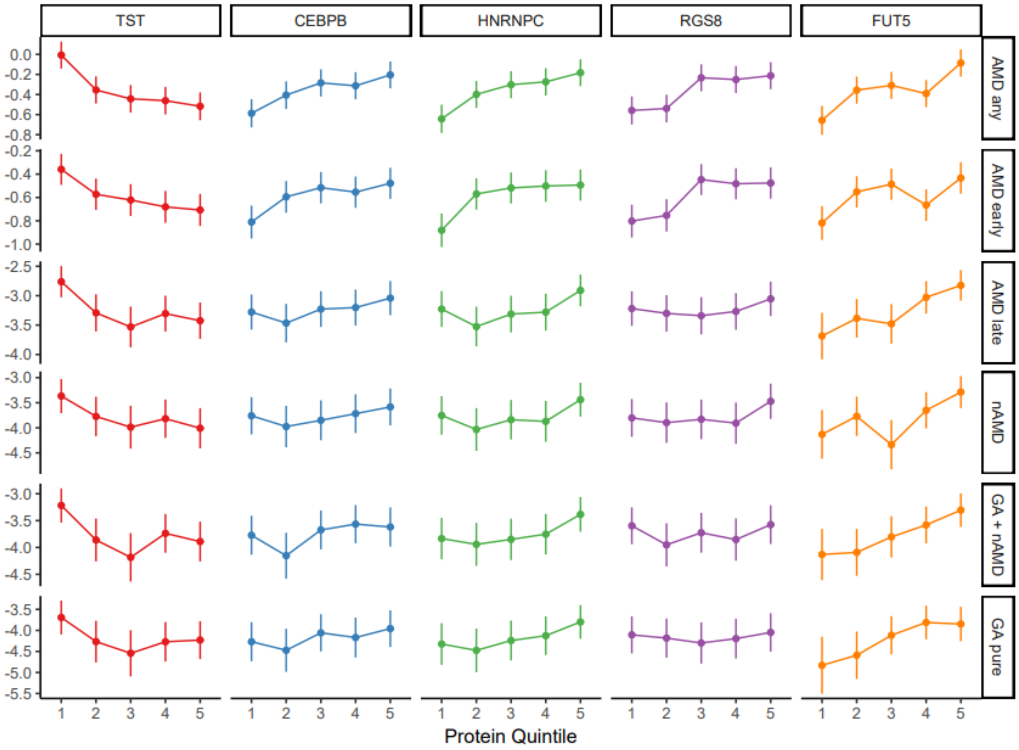
TST
CEBPB
HNRNPC
RGS8
FUT5
AMD any
AMD early
AMD late
nAMD
GA + nAMD
GA pure
Protein Quintile

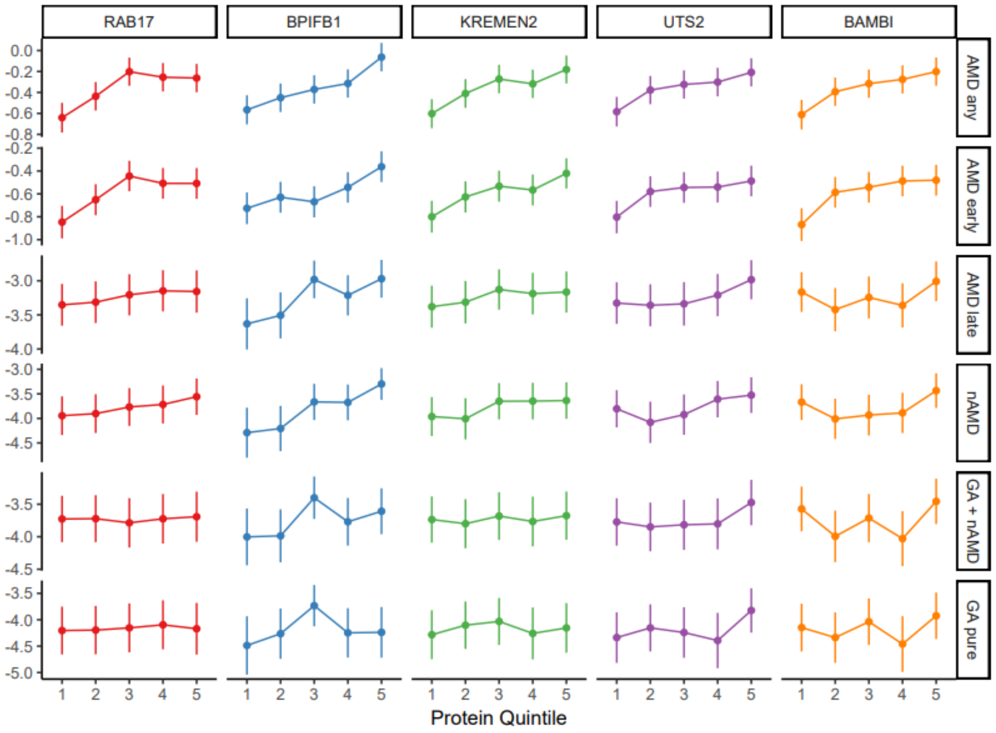
RAB17
BPIFB1
KREMEN2
UTS2
BAMBI
AMD any
AMD early
AMD late
nAMD
GA + nAMD
GA pure
Protein Quintile

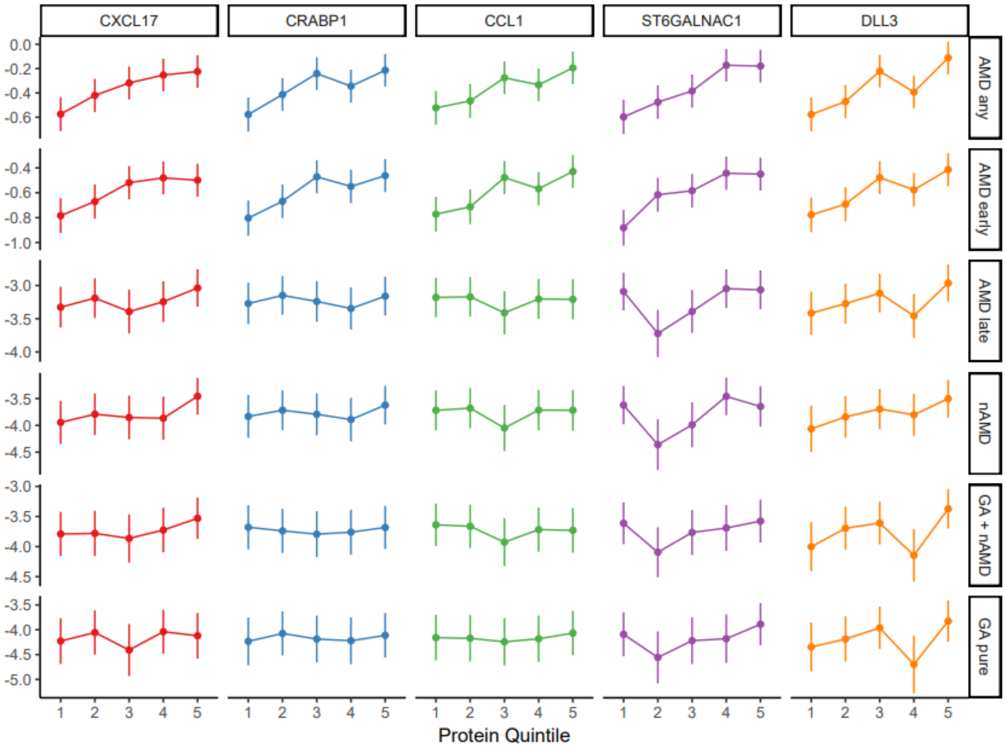
CXCL17
CRABP1
CCL1
ST6GALNAC1
DLL3
AMD any
AMD early
AMD late
nAMD
GA + nAMD
GA pure
Protein Quintile

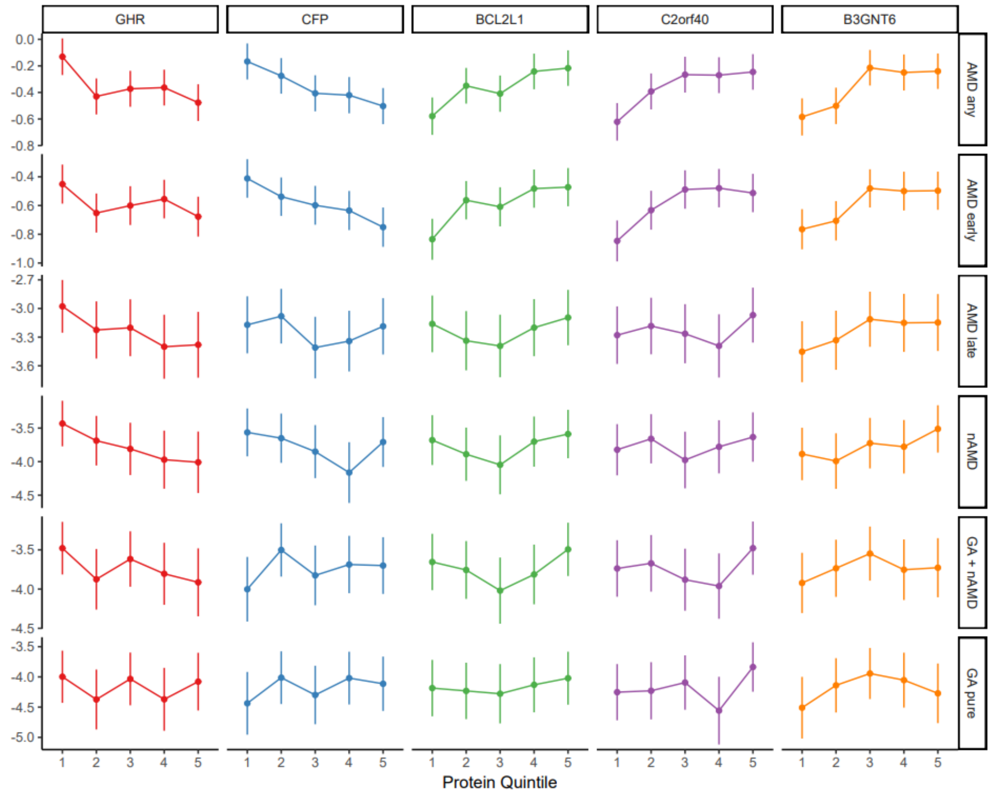
GHR
CFP
BCL2L1
C2orf40
B3GNT6
AMD any
AMD early
AMD late
nAMD
GA + nAMD
GA pure
Protein Quintile

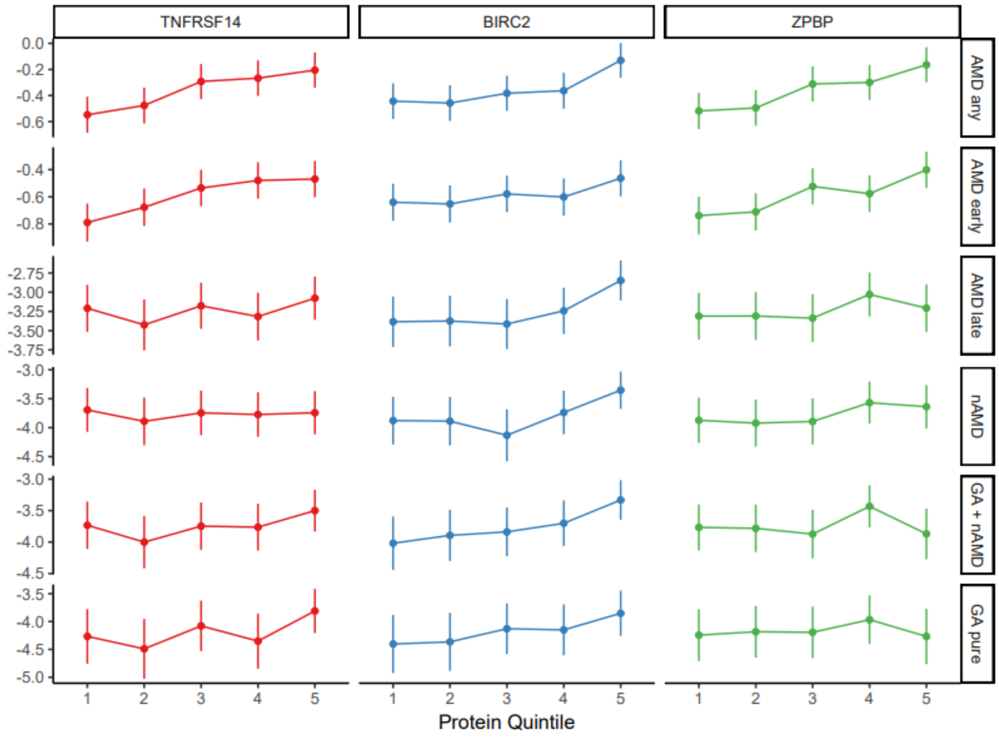
TNFRSF14
BIRC2
ZPBP
AMD any
AMD early
AMD late
nAMD
GA + nAMD
GA pure
Protein Quintile

Supplementary Fig. 3. **Quintiles of the 28 AMD-associated proteins in relation to various AMD-related outcomes**. The x-axis represents each protein's quintiles, while the y-axis represents the estimate as beta = log(OR). The center data point per each quintile represents the mean beta with error bars as the 95% confidence intervals. Protein annotations are shown in boxes at the top of each graph, while the various outcomes including AMD any, AMD early, AMD late, nAMD, GA + nAMD (GA with possibly some exudative AMD), and GA pure) are shown in boxes to the right. GA, geographic atrophy AMD. nAMD, neovascular AMD. The definition of early-stage AMD was according to Holliday et al.[20] The number of patients in each AMD-related group in the AGES-RS cohort is shown in Supplementary Data 1 and described as well in Methods.

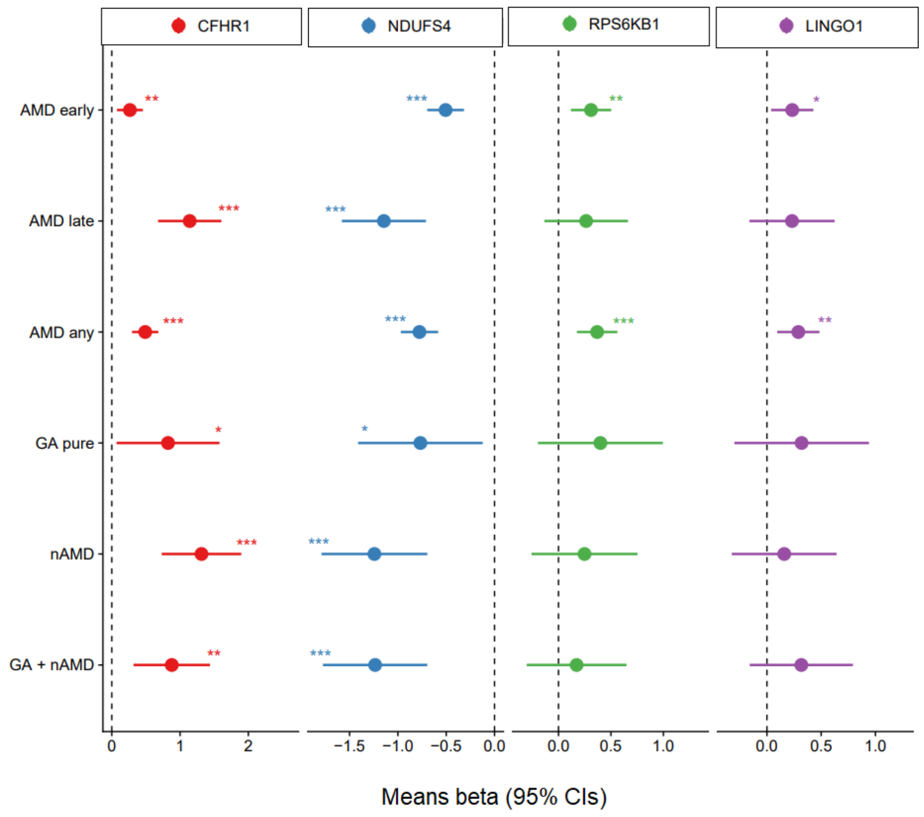
CFHR1
NDUFS4
RPS6KB1
LINGO1
AMD early
AMD late
AMD any
GA pure
nAMD
GA + nAMD
Means beta (95% CIs)

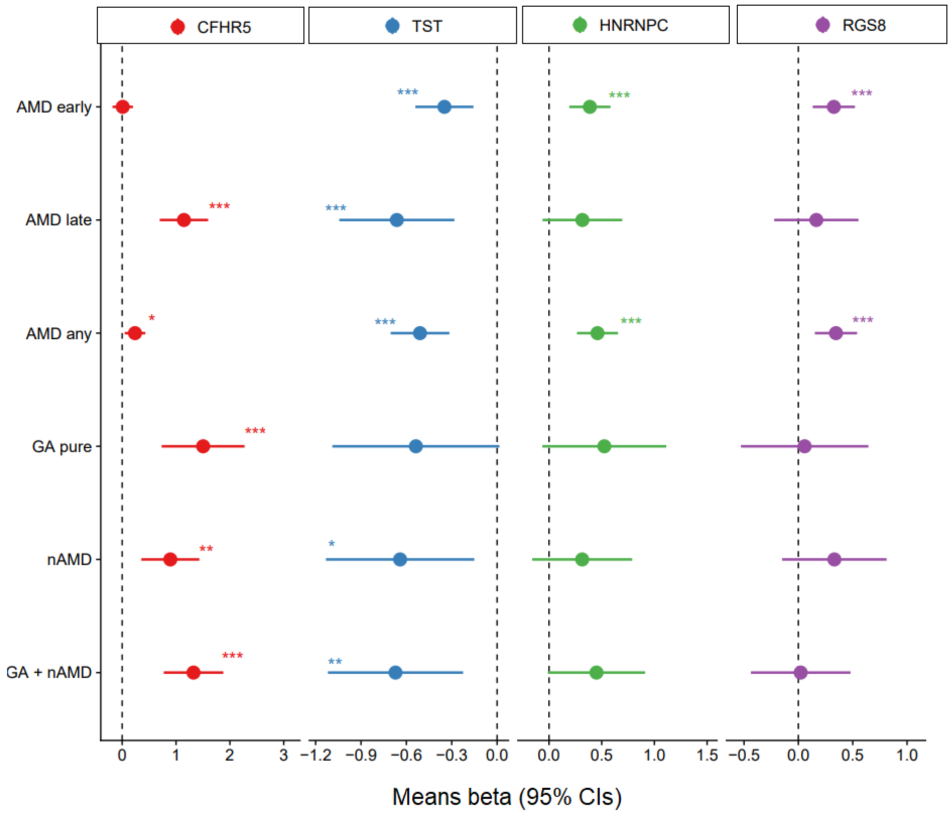
CFHR5
TST
HNRNPC
RGS8
AMD early
AMD late
AMD any
GA pure
nAMD
GA + nAMD
Means beta (95% CIs)

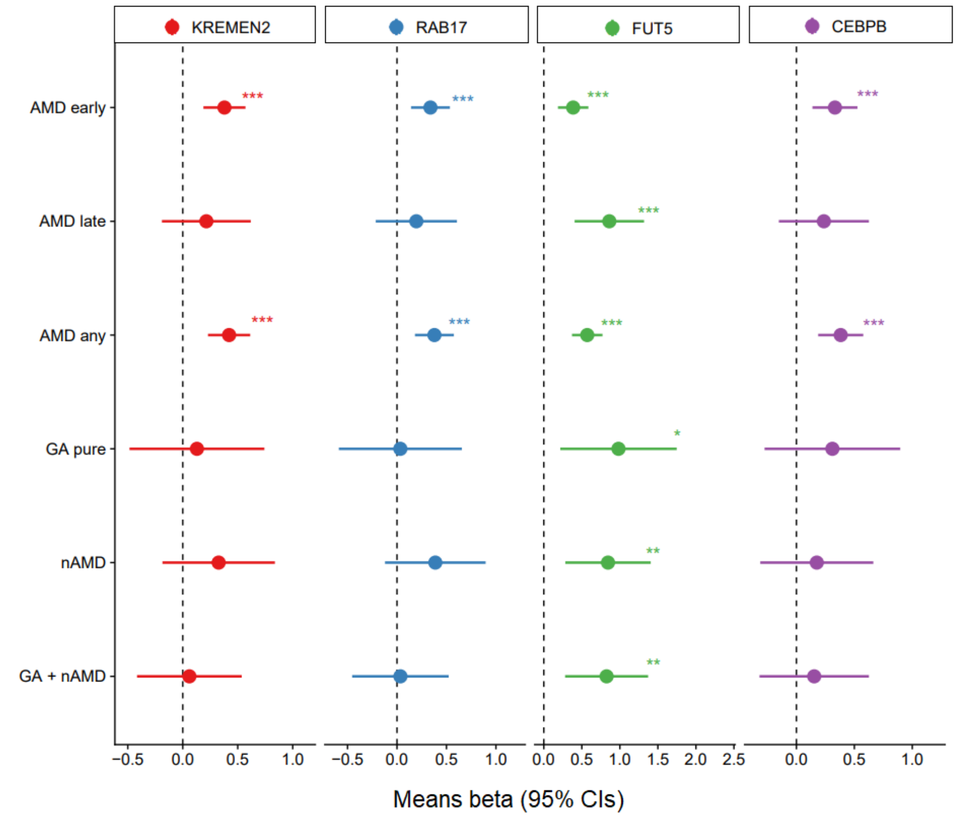
KREMEN2
RAB17
FUT5
CEBPB
AMD early
AMD late
AMD any
GA pure
nAMD
GA + nAMD
Means beta (95% CIs)

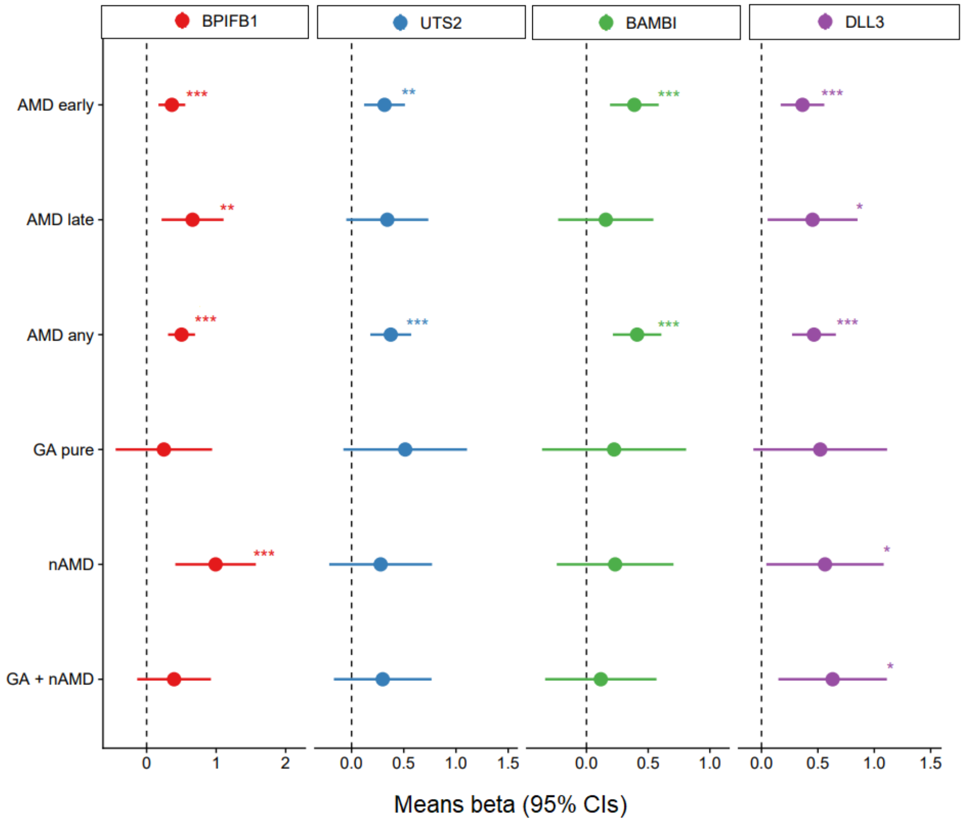
BPIFB1
UTS2
BAMBI
DLL3
AMD early
AMD late
AMD any
GA pure
nAMD
GA + nAMD
Means beta (95% CIs)

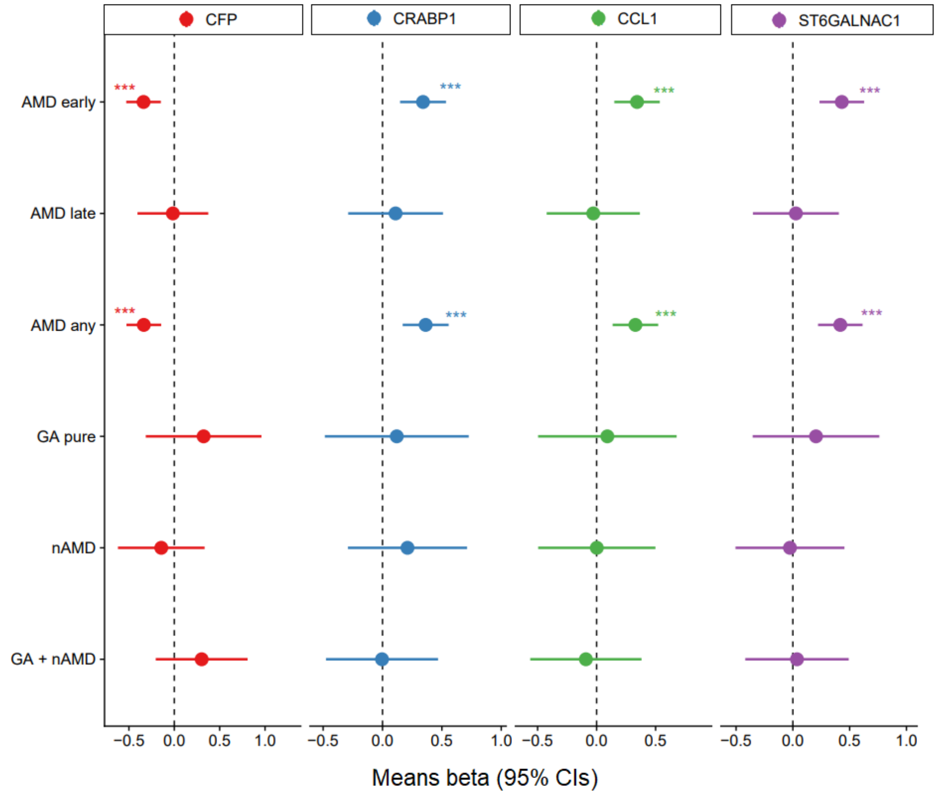
CFP
CRABP1
CCL1
ST6GALNAC1
AMD early
AMD late
AMD any
GA pure
nAMD
GA + nAMD
Means beta (95% CIs)

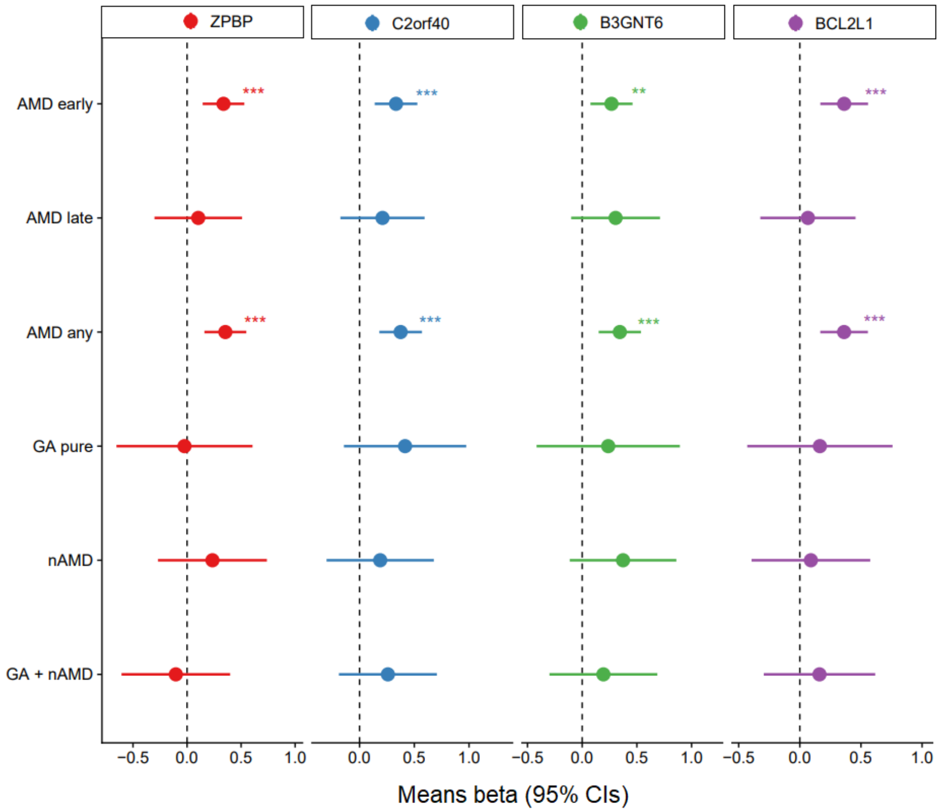
ZPBP
C2orf40
B3GNT6
BCL2L1
AMD early
AMD late
AMD any
GA pure
nAMD
GA + nAMD
Means beta (95% CIs)

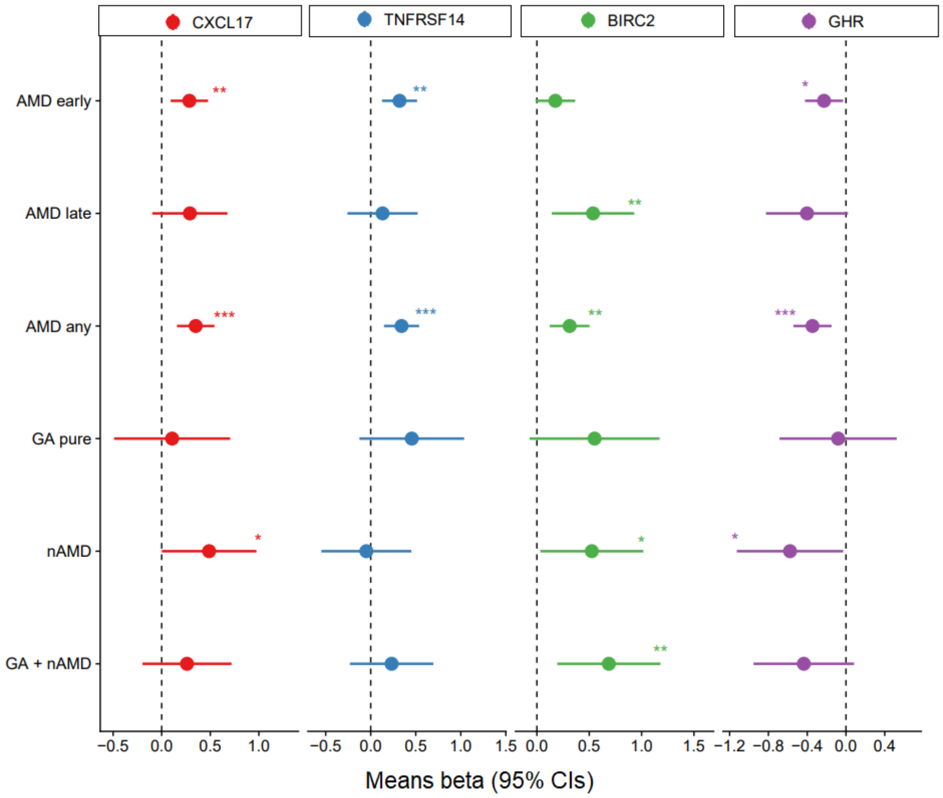

Supplementary Fig. 4. **The top versus bottom quintiles of the AMD-associated proteins in relation to AMD outcomes**. Association of the top and bottom quintiles of AMD-linked protein levels to the various AMD related outcomes (see Methods for details). The y-axis reveals the different AMD outcomes including geographic atrophy (GA/dry AMD) and neovascular AMD (nAMD), while the x-axis highlights the center data point as the mean estimate of beta in the logistic regression (logOR) using quintiles as continuous predictor variable and the error bars represent 95% CIs. ***(P-value < 0.001, two-sided), **(P-value < 0.01, two-sided), *(P-value < 0.05, two-sided). The definition of early-stage AMD was according to Holliday et al.[20] The number of patients in each AMD-related group in the AGES-RS cohort is shown in Supplementary Data 1 and described in Methods.

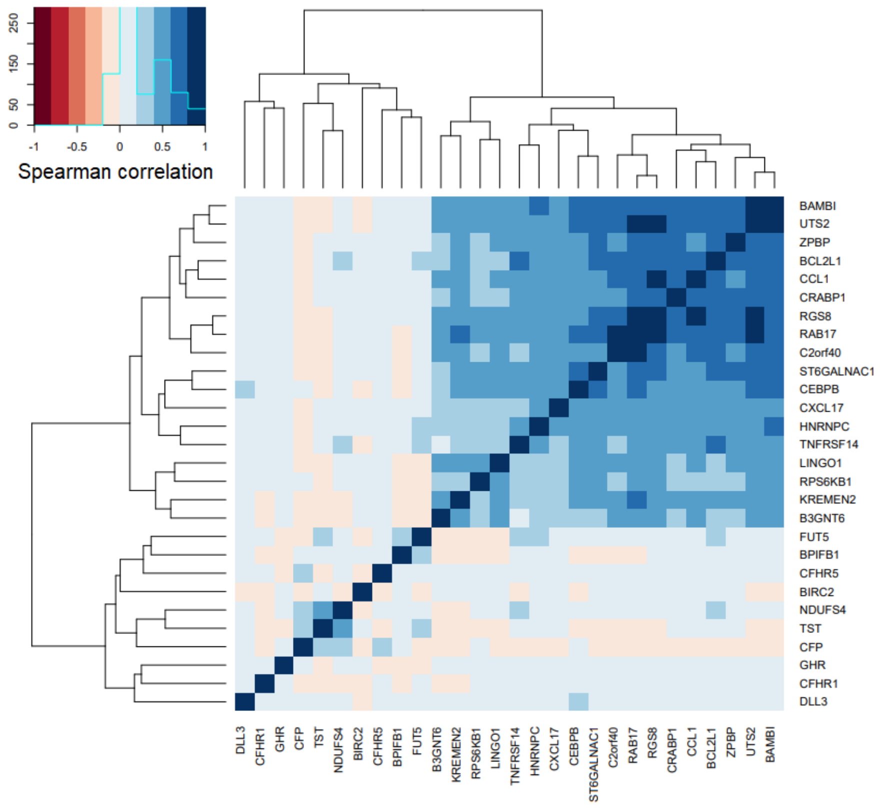

Supplementary Fig. 5. A matrix of correlations between the 28 AMD-associated proteins. A heatmap showing the Spearman rank inter-correlations (rho metrics) of all 28 AMD-associated proteins, revealing a large cluster of inter-correlated proteins.

Single cell RNA sequencing of macular RPE-choroid

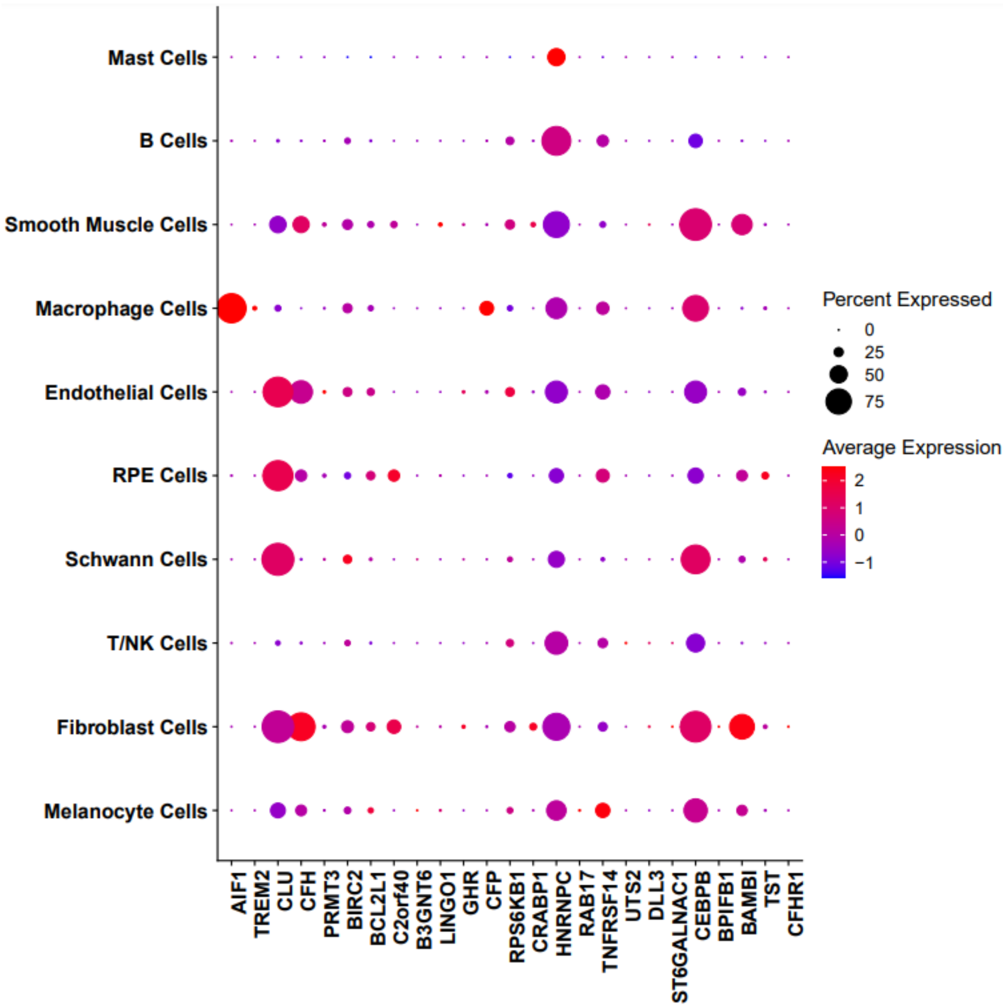

Supplementary Fig. 6. **Single cell RNA sequencing analysis of the eye**. RNA sequencing was used to examine the expression of the various AMD-associated proteins described in the current study in the RPE choroid (macula), at a single cell level, from publicly available dataset (GSE135922)[21] (see Methods for details).

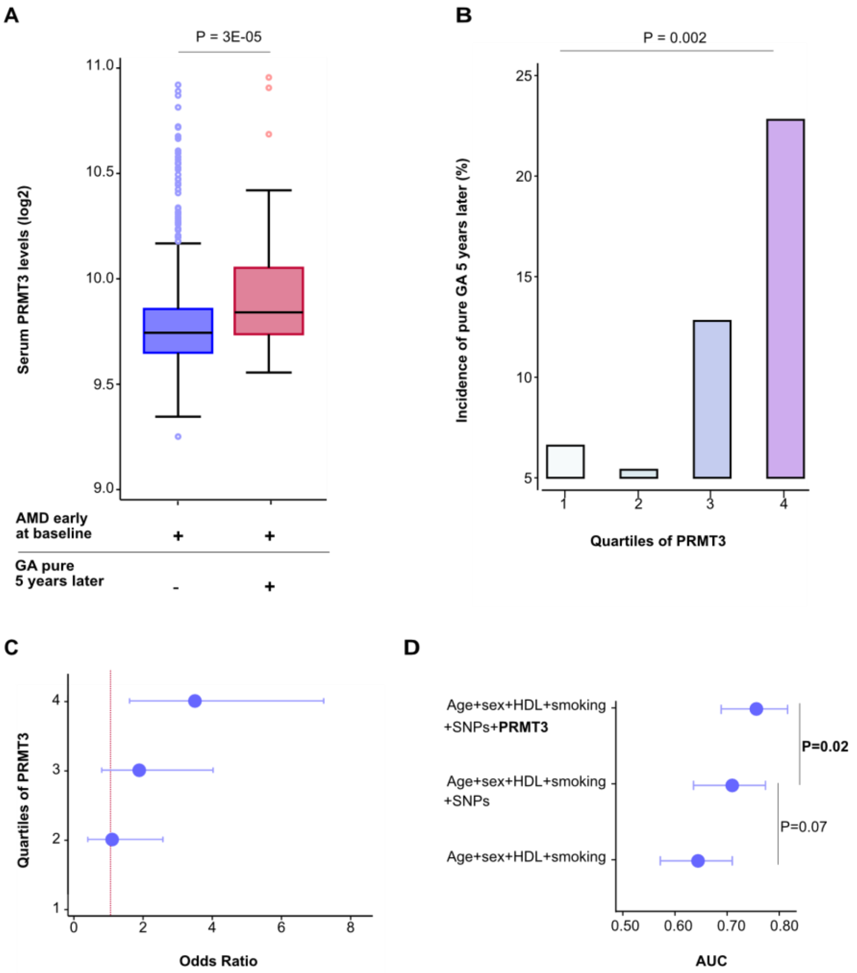

Supplementary Fig. 7. **PRMT3 is a biomarker for the progression of pure geographic atrophy**. **A**. Boxplot depicting the distribution of PRMT3 by progression status on a log2 scale. The blue box represents individuals (n = 560) who had early-stage AMD (definition by Holliday et al.[20]) at baseline and after a five-year follow-up, while the red box represents subjects (n = 64) who progressed from early-stage AMD to pure geographic atrophy (GA). The boxplot indicates median value, 25th and 75th percentiles. Whiskers extend to smallest/largest value no further than 1.5 × interquartile range with outliers being shown. The highlighted P-value is two-sided, obtained from age and sex-adjusted logistic regression analysis. **B**. The observed percentage incidence of pure GA at five-year follow-up (y-axis), as represented by PRMT3 quartiles (x-axis), was assessed using an age and sex adjusted logistic model. The P-value shown is two-sided. **C**. Age and sex adjusted odds ratios (OR) from a logistic regression model for progression to pure GA after a five-year follow-up by PRMT3 quartiles (1st quartile as reference). The F-test P-value = 0.001 is two-sided. The error bars for the mean OR (center) are the 95% CIs. **D**. Area under the curve (AUC) comparison for various logistic models with incident pure GA as the outcome variable and various predictors such as age, HDL cholesterol (HDL), smoking (current), AMD genetic risk variants (single nucleotide polymorphisms; SNPs) at chromosomes 1 (rs10922109 and rs570618) and 10 (rs3750846), and PRMT3. The error bars for the mean AUC (center) are the 95% CIs. The highlighted P-values are two-sided.

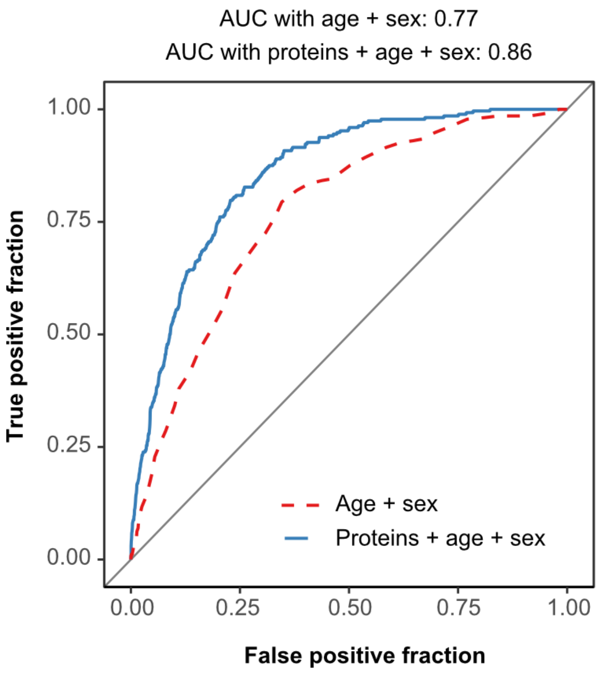

Supplementary Fig. 8. **The ability of the 21 protein predictors to classify late-stage AMD**. A receiver operating characteristic curve (ROC) for the diagnostic ability of the 21 protein predictors to classify late-stage AMD. A F-test of equality demonstrates a significant ($P = 1.4 \times 10^{-17}$, two-sided) difference the demographics (age + sex, red broken curve) versus the demographics (age + sex) plus proteins (blue curve) ROC curves.

A

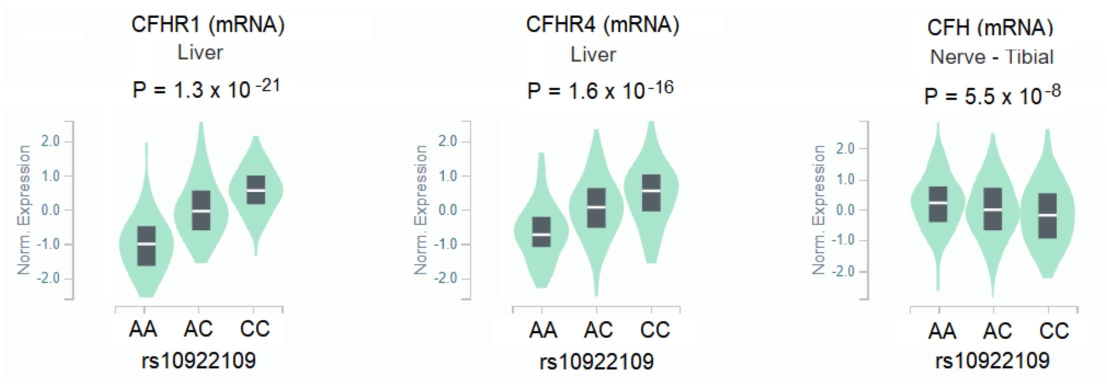

B

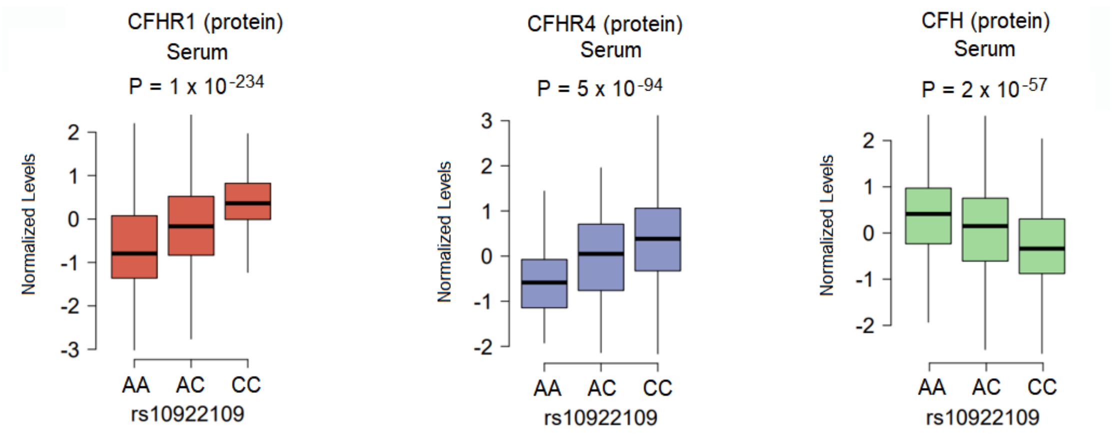

Supplementary Fig. 9. **Matching mRNA and protein changes associated with rs10922109**. **A**. Violin plots showing mRNA levels of CFHR1, CFHR4 and CFH as a function of copy C alleles for the variant rs10922109. The data came from the Genotype-Tissue Expression (GTEx) portal[22]. The tissue where the expression QTL (eQTL) was found, as well as the P-values (two-sided), are shown at the top of each plot. **B**. Box plot showing serum levels of CFHR1, CFHR4 and CFH as a function of copy C alleles for the variant rs10922109. The P-values (two-sided) at the top of each graph in **A** and **B** are the results of linear regression analyses. All box plots show median (middle line), 25th, 75th percentile (box) and 5th and 95th percentile (whiskers).

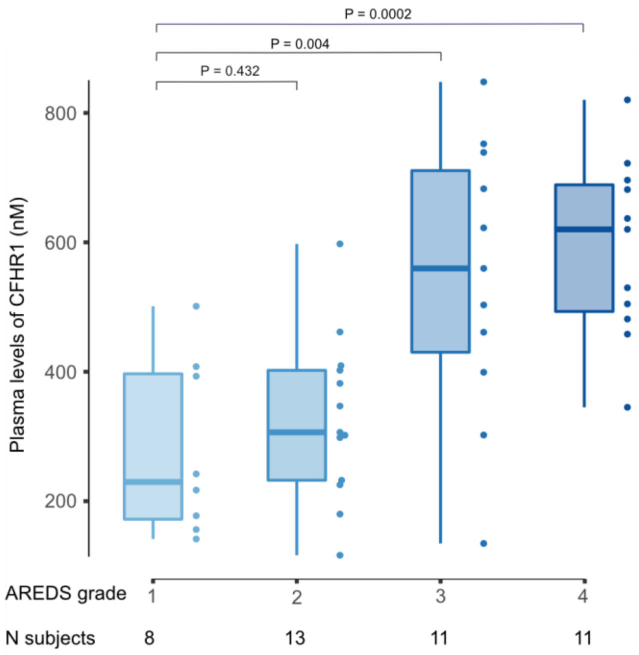

Supplementary Fig. 10. **CFHR1 levels changing from no AMD to advanced AMD.** Box plots showing increasing nM concentrations of CFHR1 from no AMD to early AMD to advanced nAMD. Here, AMD patients had AREDS grade 2-4, while AREDS grade 1 are healthy aged-matched volunteers (see Methods for details). Protein concentrations were determined by ELISA. Box plots show median (middle line), 25th, 75th percentile (box) and 5th and 95th percentile (whiskers), while individual data points are shown next to each box plot. The P-values (two-sided) at the top of each boxplot are the result of a Student´s t-test.

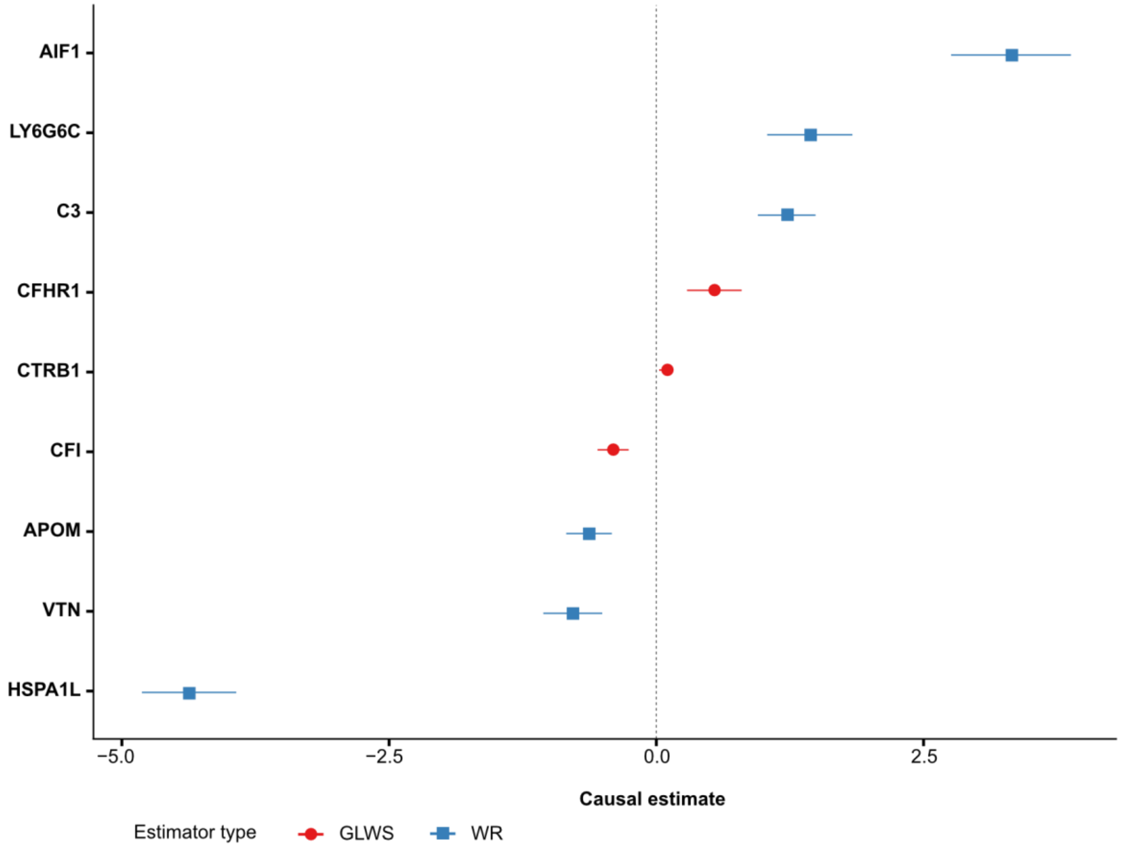

Supplementary Fig. 11. **MR analysis identifies significant protein causal candidates for AMD**. Significant (FDR < 0.05) causal estimates (center data points) and their 95% confidence intervals (error bars) for proteins with multiple genetic instruments (red data points: GWLS, generalized weighted least squares) or proteins with a single genetic instrument (blue data points: WR, Wald ratio), after adjusting the P-value (two-sided) for all 1327 aptamers with *cis*-acting variants using the Benjamini-Hochberg method. The proteins causally linked to AMD are depicted on the y-axis.

Supplementary References

1. Pool, F.M., Kiel, C., Serrano, L. & Luthert, P.J. Repository of proposed pathways and protein-protein interaction networks in age-related macular degeneration. *NPJ Aging Mech Dis* **6**, 2 (2020).
2. Rosenfeld, P.J. Lessons Learned From Avastin and OCT-The Great, the Good, the Bad, and the Ugly: The LXXV Edward Jackson Memorial Lecture. *Am J Ophthalmol* **204**, 26-45 (2019).
3. Wang, L., *et al.* Lipoprotein particles of intraocular origin in human Bruch membrane: an unusual lipid profile. *Invest Ophthalmol Vis Sci* **50**, 870-877 (2009).
4. Hollyfield, J.G., Salomon, R.G. & Crabb, J.W. Proteomic approaches to understanding age-related macular degeneration. *Adv Exp Med Biol* **533**, 83-89 (2003).
5. Rudolf, M., *et al.* Apolipoprotein A-I Mimetic Peptide L-4F Removes Bruch's Membrane Lipids in Aged Nonhuman Primates. *Invest Ophthalmol Vis Sci* **60**, 461-472 (2019).
6. Crabb, J.W., *et al.* Drusen proteome analysis: an approach to the etiology of age-related macular degeneration. *Proc Natl Acad Sci U S A* **99**, 14682-14687 (2002).
7. Curcio, C.A., Johnson, M., Huang, J.D. & Rudolf, M. Aging, age-related macular degeneration, and the response-to-retention of apolipoprotein B-containing lipoproteins. *Prog Retin Eye Res* **28**, 393-422 (2009).
8. Baek, J.H., *et al.* Quantitative proteomic analysis of aqueous humor from patients with drusen and reticular pseudodrusen in age-related macular degeneration. *BMC Ophthalmol* **18**, 289 (2018).
9. Heesterbeek, T.J., *et al.* Complement Activation Levels Are Related to Disease Stage in AMD. *Invest Ophthalmol Vis Sci* **61**, 18 (2020).
10. Alves, C.H., Fernandes, R., Santiago, A.R. & Ambrósio, A.F. Microglia Contribution to the Regulation of the Retinal and Choroidal Vasculature in Age-Related Macular Degeneration. *Cells* **9**(2020).
11. Suarez-Calvet, M., *et al.* sTREM2 cerebrospinal fluid levels are a potential biomarker for microglia activity in early-stage Alzheimer's disease and associate with neuronal injury markers. *EMBO Mol Med* **8**, 466-476 (2016).
12. Jonsson, T., *et al.* Variant of TREM2 associated with the risk of Alzheimer's disease. *N Engl J Med* **368**, 107-116 (2013).
13. Weiss, F.U., Skube, M.E. & Lerch, M.M. Chronic pancreatitis: an update on genetic risk factors. *Curr Opin Gastroenterol* **34**, 322-329 (2018).
14. Zhang, G., *et al.* Integration of metabolomics and transcriptomics revealed a fatty acid network exerting growth inhibitory effects in human pancreatic cancer. *Clin Cancer Res* **19**, 4983-4993 (2013).
15. Tamura, K., *et al.* Mutations in the pancreatic secretory enzymes CPA1 and CPB1 are associated with pancreatic cancer. *Proc Natl Acad Sci U S A* **115**, 4767-4772 (2018).
16. Teich, N., *et al.* Interaction between trypsinogen isoforms in genetically determined pancreatitis: mutation E79K in cationic trypsin (PRSS1) causes increased transactivation of anionic trypsinogen (PRSS2). *Hum Mutat* **23**, 22-31 (2004).
17. Fritsche, L.G., *et al.* A large genome-wide association study of age-related macular degeneration highlights contributions of rare and common variants. *Nat Genet* **48**, 134-143 (2016).

18. Emilsson, V., *et al.* Co-regulatory networks of human serum proteins link genetics to disease. *Science* **361**, 769-773 (2018).
19. Jonasson, F., *et al.* Five-year incidence, progression, and risk factors for age-related macular degeneration: the age, gene/environment susceptibility study. *Ophthalmology* **121**, 1766-1772 (2014).
20. Holliday, E.G., *et al.* Insights into the genetic architecture of early stage age-related macular degeneration: a genome-wide association study meta-analysis. *PLoS One* **8**, e53830 (2013).
21. Voigt, A.P., *et al.* Single-cell transcriptomics of the human retinal pigment epithelium and choroid in health and macular degeneration. *Proc Natl Acad Sci U S A* **116**, 24100-24107 (2019).
22. The GTEx Consortium atlas of genetic regulatory effects across human tissues. *Science* **369**, 1318-1330 (2020).
