# Supplementary material for: A proteogenomic signature of age-related macular degeneration in blood
Source: Nat Commun. 2022 Jun 13;13:3401. doi: 10.1038/s41467-022-31085-x (PMC9192739; doi:10.1038/s41467-022-31085-x)
Supplement: Supplementary file 3 — Description of Additional Supplementary Files [file 41467_2022_31085_MOESM3_ESM.pdf]

Description of Additional Supplementary Files

File Name: **Supplementary Data 1**
Description: Some baseline characteristics of the AGES Reykjavik study cohort: Numbers are mean(SD) for continuous-, N(%) for categorical- and median[IQR] for skewed variables. Reported P-values are two-sided. Abbreviations: SBP, systolic blood pressure; DBP, diastolic blood pressure; TOTC, total cholesterol; LDLC, LDL cholesterol; TG, triglyceride; FG, fasting blood glucose; T2D, type 2 diabetes; MetS, metabolic syndrome; CHD, coronary heart disease; AMD, age-related macular degeneration; N/A, not applicable.

File Name: **Supplementary Data 2**
Description: Logistic regression analysis of 4137 proteins for association to early, late and any AMD using Holliday et al. (PMID: 23326517) medical definition of early-stage AMD. Study-wide significant protein-to-disease associations are found at P-value < 1.2×10^{-5}. Reported P-values are two-sided. The adjusted r^2 represents the variance in outcome explained, whereas AUC represents the area under the curve calculated for each individual protein.

File Name: **Supplementary Data 3**
Description: Logistic regression analysis of 4137 proteins for association to early, late and any AMD using Jonasson et al. (PMID: 24768241) medical definition of early-stage AMD. Study-wide significant protein-to-disease associations are found at P-value < 1.2×10^{-5}. Reported P-values are two-sided. The adjusted r^2 represents the variance in outcome explained, whereas AUC represents the area under the curve calculated for each individual protein.

File Name: **Supplementary Data 4**
Description: Aptamer-based pull-down and mass spectrometry was used to validate aptamer specificity (see Methods). MRM stands for multiple reaction monitoring, and DDA stands for data-dependent acquisition.

File Name: **Supplementary Data 5**
Description: The serum protein network modules (PMID: 30072576) containing any of the 28 AMD-associated proteins listed in Supplementary Data 2 and 3. 12 out of the 28-associated proteins are over-represented (FET P = 2.6×10^{-8}, two-sided) in module PM13.

File Name: **Supplementary Data 6**
Description: Tissue distribution of RNA and/or protein expression for each of the 28 AMD-associated serum proteins using the GTEx (PMID: 32913098) and The Human Protein Atlas (PMID: 25613900) databases, respectively.

File Name: **Supplementary Data 7**
Description: All independent and genome-wide significant (P < 5×10^{-8}) variants associated with AMD in Fritsche et al. (PMID: 26691988), and for which there was a genotype information in the AGES-RS, were tested for association (linear regression) to AMD in the AGES-RS cohort and for an effect on serum protein levels. Reported P-values are two-sided. The two columns under the header "Serum proteins affected" show the number of aptamers and corresponding proteins

associated with each of the AMD-associated variants when tested against the 4782 human serum protein targets.

File Name: **Supplementary Data 8**
Description: The table includes all serum proteins associated (via linear regression) with the AMD-linked variants described by Fritsche et al. (PMID: 26691988), but excludes the trans hotspot locus rs11080055, which is shown in Supplementary Data 9. Reported P-values are two-sided. To account for multiple comparisons, significant associations with P-values of $< 1\times10^{-6}$ are reported. ↑, A1 is the risk allele; ↓, A1 is the protective allele. Inf (infinite) means that the protein encoding gene is on different chromosome than the AMD-associated variant.

File Name: **Supplementary Data 9**
Description: All serum proteins associated (via linear regression) with the trans hotspot mediator rs11080055 variant. To account for multiple comparisons, significant associations with P-values of $< 1\times10^{-6}$ are reported. Reported P-values are two-sided. ↑, A1 is the risk allele; ↓, A1 is the protective allele. Inf (infinite) means that the protein encoding gene is on different chromosome than the AMD-associated variant.

File Name: **Supplementary Data 10**
Description: Enrichment of pathways among serum proteins regulated by AMD-linked variants using g:Profiler (PMID: 31066453). The trans hotspot locus at rs11080055 was omitted from the analysis. The hypergeometric test was used to assess significance of enriched categories, adjusting for multiple testing. The reported P-values are two-sided.

File Name: **Supplementary Data 11**
Description: Individual proteins from the serum protein network module PM13 (PMID: 30072576) that are linked to AMD-linked GWAS genetic variants.

File Name: **Supplementary Data 12**
Description: Overrepresentation of proteins associated with AMD-linked genetic variants in previously identified serum protein network modules by Emilsson et al. (PMID: 30072576). The Fisher exact test (FET) was used, and the reported P-values are two-sided.

File Name: **Supplementary Data 13**
Description: Association (linear regression) of AMD-linked variants with various serum protein network modules via their Eigenproteins (Eigenproteins defined in PMID: 30072576). A $P < 1\times10^{-6}$ was considered significant for association to the Eigenproteins (1st and 2nd PCs), based on Bonferroni adjustment. Reported P-values are two-sided. We only highlight significant findings in this table. The column "Cluster" refers to the network supercluster that demonstrates module relatedness (PMID: 30072576). The PhenoScanner database (PMID: 31233103) was used to determine the relationships between network-associated AMD SNPs and various outcomes. Abbreviations for outcomes listed in the column "Outcomes previously linked to the lead SNP": RA, rheumatoid arthritis; AMD, age-related macular degeneration; MPV, mean platelet volume; TG, triglyceride; LOAD, late-onset Alzheimer´s disease; HDL, HDL cholesterol; LDL, LDL cholesterol; CRP, C-reactive protein; T2D, type two diabetes; Neovascularization, NV; Geographic atrophy; GA

File Name: **Supplementary Data 14**
Description: The association (linear regression) of the variant rs6677604 tagging the CFHR3-CFHR1 deletion to serum proteins. To account for multiple comparisons, significant associations with P-values of < 1×10^{-6} are reported. Reported P-values are two-sided. A1 is the effect allele. Inf (infinite) means that the protein encoding gene is on different chromosome than the AMD-associated variant.

File Name: **Supplementary Data 15**
Description: Except for CFHR1, a logistic regression analysis of the eight proteins highlighted in Supplementary Fig. 11 was performed for association with early, late, and any AMD using both definitions of early-stage AMD. Protein-trait associations are found to be significant at a P-value < 0.0063 (0.05/8) (colored blue). Reported P-values are two-sided. The adjusted r^2 represents the variance in outcome explained, whereas the AUC represents the calculated area under the curve for each individual protein.
