# Supplementary material for: A proteogenomic signature of age-related macular degeneration in blood
Source: Nat Commun. 2022 Jun 13;13:3401. doi: 10.1038/s41467-022-31085-x (PMC9192739; doi:10.1038/s41467-022-31085-x)
Supplement: Supplementary file 19 — Reporting Summary [file 41467_2022_31085_MOESM19_ESM.pdf]

Corresponding author(s): Valur Emilsson and Tony E Walshe

Last updated by author(s): May 7, 2022

Reporting Summary

Nature Portfolio wishes to improve the reproducibility of the work that we publish. This form provides structure for consistency and transparency in reporting. For further information on Nature Portfolio policies, see our Editorial Policies and the Editorial Policy Checklist.

Statistics

For all statistical analyses, confirm that the following items are present in the figure legend, table legend, main text, or Methods section.

n/a	Confirmed	
☐	☒	The exact sample size (*n*) for each experimental group/condition, given as a discrete number and unit of measurement
☐	☒	A statement on whether measurements were taken from distinct samples or whether the same sample was measured repeatedly
☐	☒	The statistical test(s) used AND whether they are one- or two-sided<br>*Only common tests should be described solely by name; describe more complex techniques in the Methods section.*
☐	☒	A description of all covariates tested
☐	☒	A description of any assumptions or corrections, such as tests of normality and adjustment for multiple comparisons
☐	☒	A full description of the statistical parameters including central tendency (e.g. means) or other basic estimates (e.g. regression coefficient) AND variation (e.g. standard deviation) or associated estimates of uncertainty (e.g. confidence intervals)
☐	☒	For null hypothesis testing, the test statistic (e.g. *F*, *t*, *r*) with confidence intervals, effect sizes, degrees of freedom and *P* value noted<br>*Give P values as exact values whenever suitable.*
☒	☐	For Bayesian analysis, information on the choice of priors and Markov chain Monte Carlo settings
☒	☐	For hierarchical and complex designs, identification of the appropriate level for tests and full reporting of outcomes
☐	☒	Estimates of effect sizes (e.g. Cohen's *d*, Pearson's *r*), indicating how they were calculated

Our web collection on statistics for biologists contains articles on many of the points above.

Software and code

Policy information about availability of computer code

Data collection	No software were used for data collection
Data analysis	R (3.6.0. 3.6.3), RStudio (1.1.456), R package Seurat (v.3.0.0), glmnet package for R (4.1-4).

For manuscripts utilizing custom algorithms or software that are central to the research but not yet described in published literature, software must be made available to editors and reviewers. We strongly encourage code deposition in a community repository (e.g. GitHub). See the Nature Portfolio guidelines for submitting code & software for further information.

Data

Policy information about availability of data

All manuscripts must include a data availability statement. This statement should provide the following information, where applicable:

- Accession codes, unique identifiers, or web links for publicly available datasets
- A description of any restrictions on data availability
- For clinical datasets or third party data, please ensure that the statement adheres to our policy

The custom-design Novartis SOMAscan data are available through a collaboration agreement with the Novartis Institutes for BioMedical Research (lori.jennings@novartis.com). Data from the AGES Reykjavik study are available through collaboration (AGES_data_request@hjarta.is) under a data usage agreement with the IHA. All access to data is controlled via the use of a subject-signed informed consent authorization. The time it takes to respond to requests varies depending on their nature and circumstances of the request, but it will not exceed 14 working days. All data supporting the conclusions of the paper are presented in the main text and freely available as a supplement to this manuscript (Supplementary Information and Supplementary Data 1-15). The GTEx database (https://gtexportal.org/home/) was used to obtain the eQTL data.

Field-specific reporting

Please select the one below that is the best fit for your research. If you are not sure, read the appropriate sections before making your selection.

☒ Life sciences ☐ Behavioural & social sciences ☐ Ecological, evolutionary & environmental sciences

For a reference copy of the document with all sections, see nature.com/documents/nr-reporting-summary-flat.pdf

Life sciences study design

All studies must disclose on these points even when the disclosure is negative.

Sample size	The study included all 5457 individuals from the AGES-RS study for whom protein measurements were available as well as comprehensive genotype and phenotype information. AGES-RS is a single-center prospective population-based study of highly phenotyped subjects (5764, mean age 76.6±5.6 years) and survivors of the 40-year-long prospective Reykjavik study (N=18,000). Of the 5764 AGES-RS participants 3411 attended a 5-year follow-up visit (AGES-RS II), also undergoing deep phenotyping.
Data exclusions	Individuals with missing genotype, phenotype information and protein measurements were excluded from the study
Replication	CFHR1 is an example of single test replication of a positive association of the protein with advanced AMD using different protein measurement methodologies, i.e. ELISA vs. the aptamer-based technology, and several samples of subjects with varying degree of AMD severity. Furthermore, Supplementary Data 7 highlights replication of many of the previously published genome-wide significant 52 GWAS lead SNPs for association to AMD in the AGES-RS cohort.
Randomization	The participants in this study were not randomized into experimental groups. More to the point, AGES-RS is a population-based study of survivors from the 40-year-long prospective Reykjavik study (random sample of 30,795 individuals), an epidemiologic study aimed at understanding aging in the context of gene/environment interactions by focusing on four biologic systems: vascular, neurocognitive (including sensory), musculoskeletal, and body composition/metabolism.
Blinding	Blinding was not relevant as this study did not compare experimental groups

Reporting for specific materials, systems and methods

We require information from authors about some types of materials, experimental systems and methods used in many studies. Here, indicate whether each material, system or method listed is relevant to your study. If you are not sure if a list item applies to your research, read the appropriate section before selecting a response.

Materials & experimental systems

n/a	Involved in the study	
☐	☒	Antibodies
☒	☐	Eukaryotic cell lines
☒	☐	Palaeontology and archaeology
☒	☐	Animals and other organisms
☐	☒	Human research participants
☒	☐	Clinical data
☒	☐	Dual use research of concern

Methods

n/a	Involved in the study	
☒	☐	ChIP-seq
☒	☐	Flow cytometry
☒	☐	MRI-based neuroimaging

Antibodies

Antibodies used	CFH antibody was purchased from Quidel (cat. #A255). CFHR1 capture and detection antibodies were purified from CFHR1-immunized rabbit antisera and depleted of cross-reactivity to CFH using Factor-H conjugated Sepharose 4B resin (as specified in Methods). Purified and recombinant CFH/CFHR proteins were employed to confirm selectivity.
Validation	We investigated any possible cross-reactivity of CFHR1 antibodies with the CFH and CFHR proteins. Recombinant CFH protein was obtained from Complement Technology (TX, US), and recombinant CFH and CFHR proteins were expressed in HEK cells. More specifically, anti-CFH antibodies demonstrated no cross-reactivity to CFHR proteins as measured by ELISA, whereas anti-CFHR1 antibodies showed trace cross-reactivity to CFHR2, however, with CFHR2 binding signal below the signal level of CFHR1 used to extrapolated unknown plasma levels

Human research participants

Policy information about studies involving human research participants

Population characteristics	The study included 5457 Icelandic individuals, i.e. 2330 males (mean age was 76.7 +/- 5.4 years) and 3127 females (mean

Population characteristics	age was 76.5 +/- 5.7 years), from the population based AGES-Reykjavik study. All AGES study cohort members are European Caucasians.
Recruitment	The current study did not include recruitment of study participants. The Reykjavik study originally comprised a random sample of 30,795 men and women born between 1907 and 1935 and living the Reykjavik in 1967. In 2002, the surviving individuals were invited to participate in the AGES Reykjavik study, which concluded in 2006, with a total sample size of 5764 survivors of the Reykjavik Study Cohort. For more details, see Harris et al., Am J Epidemiol 2007, 165: 1076-1087.
Ethics oversight	The AGES-RS was approved by the NBC in Iceland (approval number VSN-00-063), and by the National Institute on Aging Intramural Institutional Review Board, and the Data Protection Authority in Iceland.

Note that full information on the approval of the study protocol must also be provided in the manuscript.
